# Real-world utilization patterns and association of dd-cfDNA with biopsy and rejection

**DOI:** 10.3389/fmed.2026.1890561

**Published:** 2026-07-22

**Authors:** Ivana Dedinská, Aylin Akifova, Bilgin Osmanodja, Fabian Halleck, Eva V. Schrezenmeier, Parthenopi Avaniadi, Andrej Čereš, Matej Vnucak, Karol Granak, Julia Beck, Kirsten Bornemann-Kolatzki, Michael Oellerich, Ekkehard Schuetz, Klemens Budde

**Affiliations:** 1 Transplant-Nephrology Department, University Hospital Martin, Martin, Slovakia; 2 Internal Department I, Jessenius Faculty of Medicine, Comenius University and University Hospital Martin, Martin, Slovakia; 3 Department of Nephrology and Intensive Care, Charité Universitätsmedizin Berlin, Berlin, Germany; 4 Institute of Clinical Immunology and Medical Genetics, Jessenius Faculty of Medicine, Comenius University and University Hospital Martin, Martin, Slovakia; 5 Chronix Biomedical GmbH, Göttingen, Germany; 6 Department of Clinical Pharmacology, George–August University, Göttingen, Germany; 7 Insight Molecular Diagnostics, Nashville, TN, United States

**Keywords:** acute rejection, biomarker, biopsy, donor-derived cell free DNA, kidney transplantation

## Abstract

**Background:**

Donor-derived cell-free DNA (dd-cfDNA) is a noninvasive biomarker for allograft injury in kidney transplant recipients. This single-center study aimed to evaluate the clinical indications for dd-cfDNA testing, its association with biopsy findings, and the comparative utility of dd-cfDNA expressed as a percentage versus absolute copy number.

**Methods:**

A retrospective analysis was conducted of 571 dd-cfDNA measurements from 313 kidney transplant recipients at the Charité Berlin Transplant Center from May 2020 to May 2025. The clinical indications for testing, biopsy, and rejection predictors were assessed using multivariable logistic regression and linear regression.

**Results:**

Elevated creatinine (29%), elevated urine albumin-to-creatinine ratio (UACR, 10%), donor-specific antibody positivity (10%), history of rejection (9%), immunosuppression reduction (5%), and combined reasons (38%) were the most frequent indications for dd-cfDNA testing. Elevated UACR was the only independent predictor of biopsy (odds ratio [OR], 3.02; 95% confidence interval [CI], 1.39–6.58; *p* = 0.005). In the biopsy cohort, the dd-cfDNA percentage (OR, 5.46; 95% CI, 2.89–13.70; *p* = 0.0035) and copies/mL (OR, 7.05; 95% CI, 1.01–21.09; *p* = 0.0062) independently predicted rejection. ROC analysis indicated good discrimination of rejection for both copies/mL (AUC, 0.781) and percentage (AUC, 0.820). DCA revealed that both dd-cfDNA formats offered greater net clinical benefits for rejection prediction than the conventional markers.

**Conclusion:**

Although biopsy decisions primarily rely on conventional clinical parameters, dd-cfDNA can independently identify rejection and improve decision-making when integrated into multimodal post-transplant monitoring strategies.

## Introduction

Donor-derived cell-free DNA (dd-cfDNA) has garnered attention among transplant physicians as a noninvasive marker of allograft injury. Unlike conventional measures, including serum creatinine or proteinuria, dd-cfDNA directly indicates allograft cell injury and may increase before the appearance of clinical or histological signs of rejection appear (1–5). dd-cfDNA exhibits greater specificity and diagnostic accuracy for antibody-mediated rejection (ABMR) than for T-cell–mediated rejection (TCMR). Several multicenter studies and meta-analyses have demonstrated that dd-cfDNA levels consistently increase with ABMR, reflecting alloantibody-induced endothelial injury and microvascular inflammation (2–5). In contrast, the sensitivity of dd-cfDNA for TCMR is diverse and generally low, particularly for Banff grade IA–IB cellular rejection (2) and borderline changes, where focal cellular infiltrates and limited parenchymal injury may generate only modest or transient increases in dd-cfDNA (3, 6–8). dd-cfDNA elevations may also occur with non-immune causes of injury (infection, ischemia–reperfusion injury, surgical complications, or immunosuppressive states), which can result in false-positive results (9). Inter-assay variability and the lack of standardized thresholds further complicate interpretation (10).

Cost, variable reimbursement, platform turnaround times, and the need for local validation and staff training constrain implementation; accordingly, several centers have adopted targeted rather than universal use (11). Evidence gaps remain, specifically the absence of randomized or prospective outcome trials demonstrating that dd-cfDNA-guided management decreases biopsy rates and enhances long-term graft survival, as well as limited cost-effectiveness data across diverse populations and immunosuppressive regimens (3, 10).

Surveillance in stable recipients to detect subclinical allograft injury (typically monthly to quarterly during the first year and less frequently thereafter), “for-cause” testing for graft dysfunction or new proteinuria, adjunctive testing when *de novo* donor-specific antibodies (dnDSAs) are detected to assess for concurrent active injury suggestive of ABMR, and serial monitoring to assess response to anti-rejection therapy are typical clinical indications for dd-cfDNA testing (12–14). Furthermore, dd-cfDNA has recently been successfully employed as a companion biomarker in therapeutic monitoring of chronic/active ABMR treated with anti-CD38 monoclonal antibodies (daratumumab or felzartamab), offering an additional dimension to its clinical applicability (15–17).

Although the diagnostic performance of dd-cfDNA has been extensively validated, there is a paucity of systematic real-world data describing how dd-cfDNA is used in daily clinical practice, how testing indications are selected, and how results truly influence biopsy decision-making across heterogeneous patient populations. To our knowledge, no large-scale study has comprehensively analyzed real-world testing indications, clinical triggers, subsequent biopsy behavior, and biopsy-proven outcomes within a single integrated framework.

The primary aim of this study was to characterize the real-world clinical indications for donor-derived cell-free DNA (dd-cfDNA) testing in kidney transplant recipients in routine clinical practice. Secondary aims were to evaluate the clinical, biochemical, and immunological factors associated with elevated dd-cfDNA levels, to determine which testing indications were followed by kidney biopsy, and to identify parameters associated with biopsy-proven rejection. We hypothesized that biopsy decisions in routine clinical practice are primarily driven by conventional clinical markers of graft dysfunction rather than by dd-cfDNA results alone, whereas dd-cfDNA, expressed as both percentage and absolute copy number, would demonstrate stronger discrimination for biopsy-proven rejection than conventional laboratory parameters such as serum creatinine or albuminuria.

## Materials and methods

To identify the clinical indications for dd-cfDNA testing and their association with biopsy and biopsy-proven rejection, we conducted a systematic retrospective study at the Kidney Transplant Center of Charité–Universitätsmedizin Berlin, following the introduction of dd-cfDNA testing in 2020. In this study, “real-world” refers to the use of dd-cfDNA as part of routine clinical care, with clinician-directed, non-protocol ordering and interpretation rather than testing mandated by a research protocol or fixed algorithm.

Of the 1,079 dd-cfDNA measurements performed between April 29, 2020, and May 15, 2025, the following were excluded: (i) multiorgan transplant recipients, (ii) patients enrolled in interventional dd-cfDNA-related clinical trials with protocol-mandated testing, and (iii) measurements with missing key clinical data required for indication classification or outcome adjudication. Interventional-trial cases were excluded because dd-cfDNA testing in that setting was protocol-driven and, therefore, did not reflect routine clinical indications. The final analytic cohort included 571 measurements from 313 kidney only recipients. Compared with the included cohort, excluded measurements were more likely to originate from ABMR-focused interventional studies, and therefore showed higher DSA positivity, worse graft function, and higher dd-cfDNA values (Supplementary Table S1). The CONSORT-style flow diagram is shown in Supplementary Figure S1. The possibility of non-random missingness and selection bias was acknowledged.

**Figure 1:**
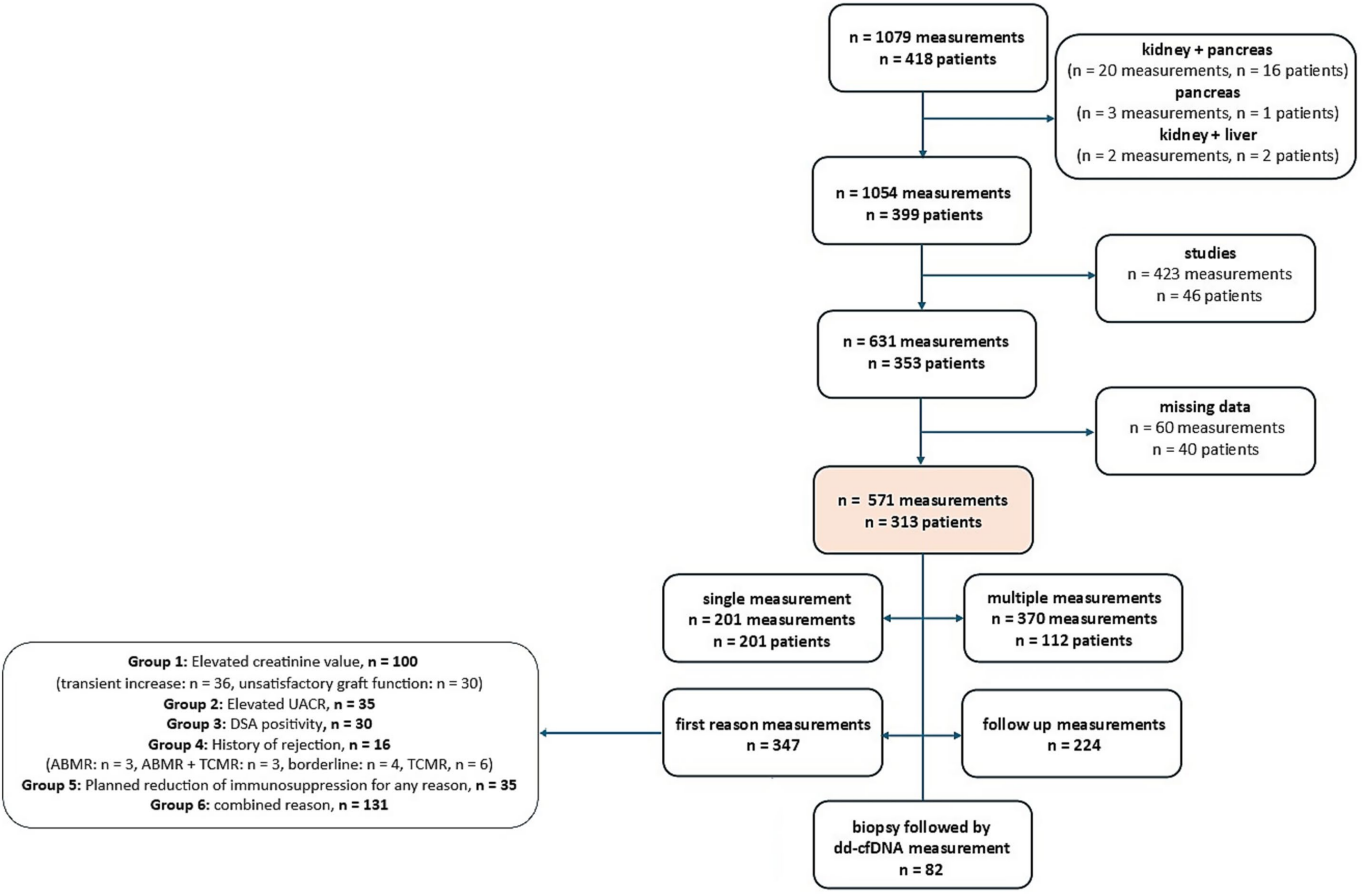
Patients and measurements evaluation. Flowchart illustrating patient selection and the classification of donor-derived cell-free DNA (dd-cfDNA) measurements included in the study. A total of 1,079 dd-cfDNA measurements from 418 transplant recipients were initially screened. Measurements from recipients of combined kidney-pancreas, pancreas, or kidney-liver transplants were excluded, as were measurements obtained within interventional studies and those with missing clinical data, resulting in a final cohort of 571 dd-cfDNA measurements from 313 kidney transplant recipients. Measurements were further categorized according to whether patients contributed a single or multiple measurements and whether the analysis included the first measurement or follow-up measurements. Clinically indicated kidney biopsies were performed after 82 dd-cfDNA measurements. First dd-cfDNA measurements were classified according to the primary indication for testing: Group 1, elevated serum creatinine; Group 2, elevated urinary albumin-to-creatinine ratio (UACR); Group 3, donor-specific antibody (DSA) positivity; Group 4, history of rejection; Group 5, planned immunosuppression reduction; and Group 6, combined indication (≥2 concurrent indications).

For patients with multiple measurements, only the first test was used to assign the clinical indication; subsequent measurements within 6 months were classified as follow-up tests. Because some recipients contributed to more than one dd-cfDNA test, observations were not statistically independent; sensitivity analyses restricted to the first test per patient yielded similar results. For each dd-cfDNA measurement, baseline serum creatinine level and UACR were defined as the mean of 3–5 preceding values. The clinical indications for testing were categorized into six predefined groups (Figure 1).

Clinical indications were not prospectively coded in the ordering system and were therefore reconstructed retrospectively through a structured review of electronic medical records. In cases with more than one plausible indication, the primary indication documented by the treating clinician was assigned; uncertain cases were excluded from indication-specific analyses. To address heterogeneity, “combined reasons” were further unpacked, and testing was also grouped according to clinical intent (for-cause vs. monitoring), as described in the Supplementary material.

Both total cfDNA and dd-cfDNA were quantified using a single-nucleotide polymorphism (SNP)-based droplet digital PCR assay, and the results are expressed as relative fractions (%) and absolute concentrations (copies/mL). For histological analyses, biopsy-proven TCMR, ABMR, and mixed rejection were classified as rejection; borderline changes were excluded; and all other diagnoses were classified as non-rejection.

Throughout the study period, no protocol (time-driven) biopsies were performed and all biopsies were clinically indicated. The dd-cfDNA results were generally available to clinicians when biopsy decisions were made. The dd-cfDNA was not embedded in a mandatory biopsy algorithm, and no predefined dd-cfDNA threshold was mandated or precluded biopsy. Diagnostic performance analyses (AUC, thresholds, sensitivity/specificity, and decision curve analysis) were therefore performed in the subset of tests followed by biopsy and reflected test performance conditional on biopsy, acknowledging potential partial verification bias.

A detailed description of patient selection, indication definitions, dd-cfDNA quantification, statistical methodology, and all sensitivity analyses is provided in the Supplementary material (Section 1A).

### Statistical analysis

Statistical analyses were performed using MedCalc version 23.3.7 (Ostend, Belgium). Descriptive statistics are presented as mean ± SD or median (IQR, 25th–75th percentile) for continuous variables and as frequencies and percentages for categorical variables. Normality was assessed using the Kolmogorov–Smirnov test. Between-group comparisons were performed using Student’s *t*-test or Mann–Whitney *U* test, as appropriate. Categorical variables were compared using the chi-square or Fisher’s exact tests. Comparisons across multiple groups were performed using ANOVA or the Kruskal–Wallis test.

Receiver operating characteristic (ROC) curve analysis was used to assess the diagnostic performance of dd-cfDNA in predicting biopsy and rejection. Optimal cutoff values were determined using the Youden index, and the AUCs were compared using the DeLong test. Multivariable logistic regression adjusted for age, sex, time post-transplant, baseline creatinine, and UACR was used to identify predictors of biopsy and rejection. Multivariable linear regression with the same adjustments was used to assess the association between dd-cfDNA levels and testing indications. Decision curve analysis was performed to evaluate the clinical net benefit of dd-cfDNA compared to baseline creatinine and UACR. Statistical significance was set at *p* < 0.05.

A more detailed description of the complete statistical analysis is provided in Supplementary material (Section 1B).

### Ethical approval

All procedures involving human participants were approved according to the ethical standards of the institutional research committee, including the 1964 Helsinki Declaration and its later amendments of comparable ethical standards.

Clinical and research activities are reported in alignment with the Principles of the Declaration of Istanbul, as outlined in the Declaration of Istanbul on Organ Trafficking and Transplant Tourism.

## Results

### Study population

After applying the exclusion criteria, 571 dd-cfDNA measurements from 313 kidney transplant recipients from May 2020 to May 2025 were analyzed. Baseline cohort characteristics are summarized in Table 1. The baseline characteristics of patients with single versus multiple dd-cfDNA measurements as well as group characteristics according to the time post-transplantation at which the measurement was performed (≤12, 12–60, and >60 months post-transplantation) are summarized in Supplementary Tables S2, S3.

**Table 1:** Basic group characteristics.

All measurements (*n* = 571)	Variable
Sex: male (%)	*N* = 209 (66.8%)
Age at kidney transplantation (years) ± SD	42.9 ± 15.3 median, 43 min, 5; max, 84
Measurements with DSA positivity at dd-cfDNA testing (%)	*N* = 109 (19.1)
Time post-kidney transplantation when dd-cfDNA was performed, median (IQR, 25th–75th percentile)	71 (IQR, 20–145)
Total cfDNA (cp/mL), median (IQR, 25th–75th percentile)	4,307 (IQR, 2,726.5–8,254.5)
dd-cfDNA (%), median (IQR, 25th–75th percentile)	0.35 (IQR, 0.2–0.85)
dd-cf-DNA (cp/mL), median (IQR, 25th–75th percentile)	16 (IQR, 8–44)
Baseline creatinine (mg/dL), median (IQR, 25th–75th percentile)	1.88 (IQR, 1.53–2.44)
Baseline UACR (mg/g), median (IQR, 25th–75th percentile)	79 (IQR, 27.3–324.5)
Biopsy following dd-cfDNA measurement (%)	n = 82 (14.4%)
- if yes, time after measurement (months), median (IQR, 25th–75th percentile)	1 (IQR, 0–4)

SD, standard deviation; IQR, interquartile range; DSA, donor-specific antibodies; dd-cfDNA, donor-derived cell-free DNA; UACR, urinary albumin-to-creatinine ratio.

Data are presented as mean ± standard deviation (SD), median (interquartile range [IQR]), or number (%), as appropriate.

### Indications for donor-derived cell-free DNA testing

**Figure 2:**
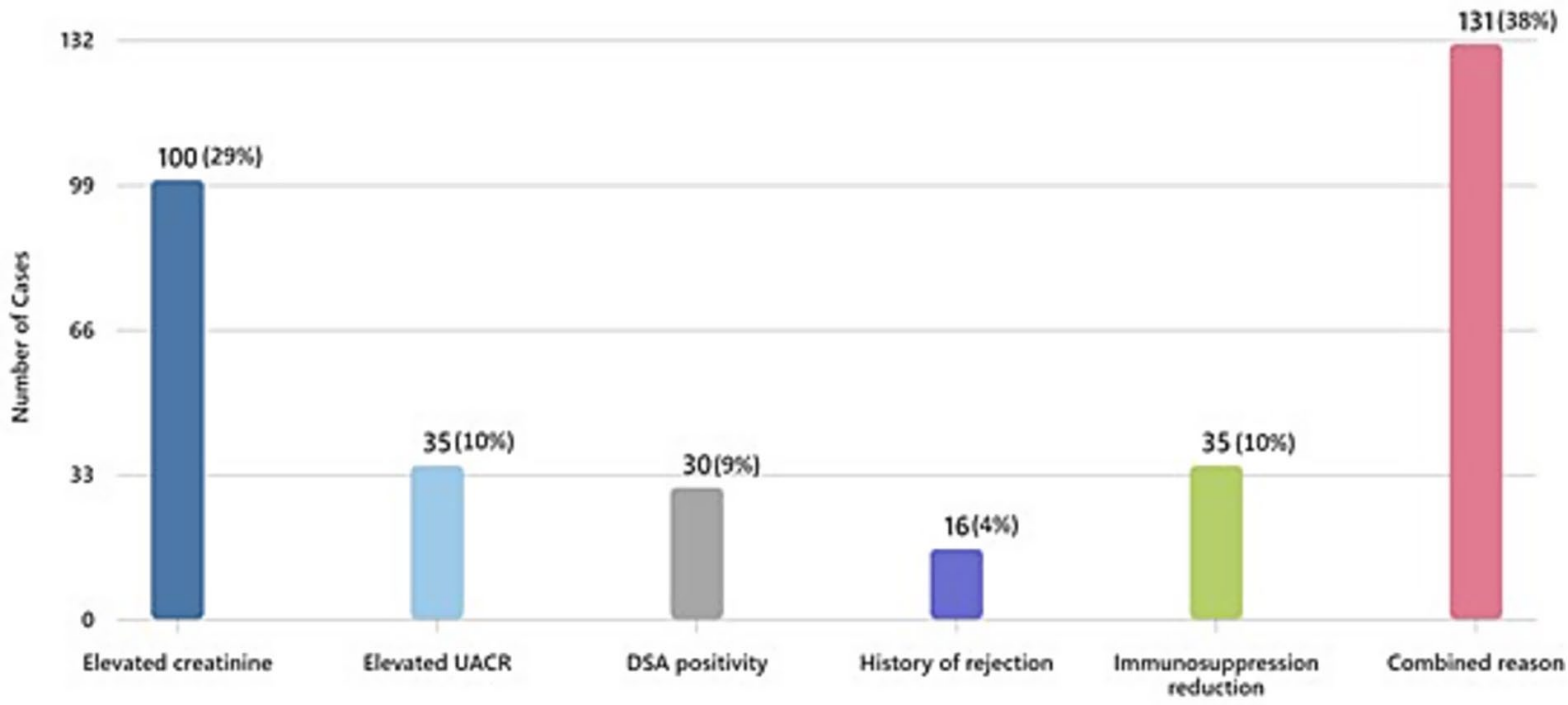
Reasons for first dd-cfDNA measurements. UACR – urinary albumin-creatinine ratio, DSA – donor specific antibodies. Distribution of the primary clinical indications leading to the first donor-derived cell-free DNA (dd-cfDNA) measurement among 347 first measurements included in the study. The most frequent indication was combined indication (131 measurements, 38%), followed by an elevated serum creatinine (100 measurements, 29%). Other indications included elevated urinary albumin-to-creatinine ratio (UACR; 35 measurements, 10%), donor-specific antibody (DSA) positivity (30 measurements, 9%), planned immunosuppression reduction (35 measurements, 10%), and history of rejection (16 measurements, 4%). UACR, urinary albumin-creatinine ratio; DSA, donor specific antibodies.

The clinical reasons for the dd-cfDNA measurements are illustrated in Figure 2. The indications included elevated serum creatinine levels, elevated UACR values, DSA positivity, history of rejection, planned IS reduction, and combined reasons (groups 1–6, respectively). The distribution of rejection phenotypes among patients with a history of rejection (group 4) is depicted in Supplementary Figure S2. Additional analyses comparing single versus multiple dd-cfDNA measurements and stratifications by time since transplantation are provided in Supplementary Figures S3–S11.

### Group comparisons

The patient and measurement characteristics stratified by indication are presented in Table 2. Significant differences were noted across the groups in terms of time since transplantation (*p* = 0.005), dd-cfDNA percentages (*p* = 0.003), dd-cfDNA copies/mL (*p* = 0.008), baseline creatinine level (*p* < 0.0001), and baseline UACR value (p < 0.0001). The combined-reason group most frequently underwent follow-up biopsies (26.7%), whereas the patients with planned IS reduction exhibited the lowest biopsy rate (5.7%).

**Table 2:** Group characteristics based on the reason for dd-cfDNA measurement.

Patients = 313 Measurements = 347	Elevated creatinine *n* = 92 *n* = 100	Elevated UACR *n* = 34 *n* = 35	DSA positivity *n* = 28 *n* = 30	History of rejection *n* = 12 *n* = 16	Reduction of immunosuppression *n* = 34 *n* = 35	Combined reasons *n* = 113 *n* = 131	*p*-value
Sex: male (%)	*n* = 61 (66.3%)	*n* = 25 (73.5%)	*n* = 16 (57.1%)	*n* = 9 (75%)	*n* = 21 (61.7%)	*n* = 77 (68.1%)	0.5699
Age at kidney transplantation (years) ± SD	45 ± 14.9 median, 46 min, 13; max, 71	43.9 ± 16.6 median, 43.5 min, 5; max, 79	42.2 ± 14 median, 41 min, 22; max, 65	44.7 ± 22.5 median, 44 min, 11; max, 84	46.2 ± 13.9 median, 47.5 min, 17; max, 71	40.8 ± 14.9 median, 40.5 min, 11; max, 81	0.3255
Time post-kidney transplantation when dd-cfDNA was performed (months), median (IQR, 25th–75th percentile)	Median, 43 (IQR, 7.75–130)	Median, 115.5 (IQR, 39.5–224.75)	Median, 73 (IQR, 35.25–153)	Median, 41.5 (IQR, 19.5–92.75)	Median, 108 (IQR, 36–166)	Median, 92 (IQR, 39.5–156.25)	**0.0050**
Total cfDNA (cp/mL), median (IQR, 25th–75th percentile)	Median, 3,911 (IQR, 2,672–6,746)	Median, 5,179 (IQR, 3,160–11,662)	Median, 3,601 (IQR, 2,543–7,975)	Median, 1,016 (IQR, 2,511–10,263)	Median, 4,179 (IQR, 2,507–6,756)	Median, 3,894 (IQR, 2,647–7,104)	0.8540
dd-cfDNA (%), median (IQR, 25th–75th percentile)	Median, 0.26 (IQR, 0.18–0.46)	Median, 0.27 (IQR, 0.19–0.37)	Median, 0.31 (IQR, 0.21–0.66)	Median, 0.26 (IQR, 0.18–0.4)	Median, 0.2 (IQR, 0.15–0.58)	Median, 0.38 (IQR, 0.2–1.25)	**0.0030**
dd-cf-DNA (cp/mL), median (IQR, 25th–75th percentile)	Median, 11 (IQR, 7–25)	Median, 13 (IQR, 7–26)	Median, 16 (IQR, 7–44)	Median, 11.5 (IQR, 7.5–50.5)	Median, 8 (IQR, 7–14)	Median, 20.5 (IQR, 8–48.25)	**0.0081**
Baseline creatinine level (mg/dL), median (IQR, 25th–75th percentile)	Median, 1.9 (IQR, 1.54–2.42)	Median, 1.67 (IQR, 1.24–2.2)	Median, 1.59 (IQR, 1.31–1.89)	Median, 2.14 (IQR, 1.81–2.35)	Median, 1.4 (IQR, 1.15–1.6)	Median, 1.9 (IQR, 1.59–2.43)	**<0.0001**
Baseline UACR value (mg/g), median (IQR, 25th–75th percentile)	Median, 47.7 (IQR, 26.9–152)	Median, 191 (IQR, 83.3–423)	Median, 35 (IQR, 11.5–93)	Median, 43.5 (IQR, 31–66.8)	Median, 24.3 (IQR, 9.9–47.9)	Median, 169 (IQR, 26.5–565.3)	**<0.0001**
Biopsy following dd-cfDNA (%)	*n* = 16 (16%)	*n* = 7 (20%)	*n* = 4 (13.3%)	*n* = 3 (18.8%)	*n* = 2 (5.7%)	*n* = 35 (26.7%)	0.0638
- if yes, time after measurement (months), median (IQR, 25th–75th percentile)	Median, 3 (IQR, 0.5–6.5)	Median, 1 (IQR, 1–5.5)	Median, 1 (IQR, 0.75–1)	Median, 4 (IQR, 2–4.5)	Median, 1.5 (IQR, 0.75–2.25)	Median, 1 (IQR, 0.25–1)	0.2094

SD, standard deviation; IQR, interquartile range; DSA, donor-specific antibodies; dd-cfDNA, donor-derived cell-free DNA; UACR, urinary albumin-to-creatinine ratio.

Data are presented as mean ± standard deviation (SD), median (interquartile range [IQR]), or number (%), as appropriate. *p*-values were calculated using the chi-square or Fisher’s exact test for categorical variables, one-way analysis of variance (ANOVA) for age at kidney transplantation, and the Kruskal–Wallis test for non-normally distributed continuous variables. Combined indication was defined as the presence of ≥2 concurrent clinical indications for dd-cfDNA testing. Bold values indicate statistically significant results (*p* < 0.05).

**Figure 3:**
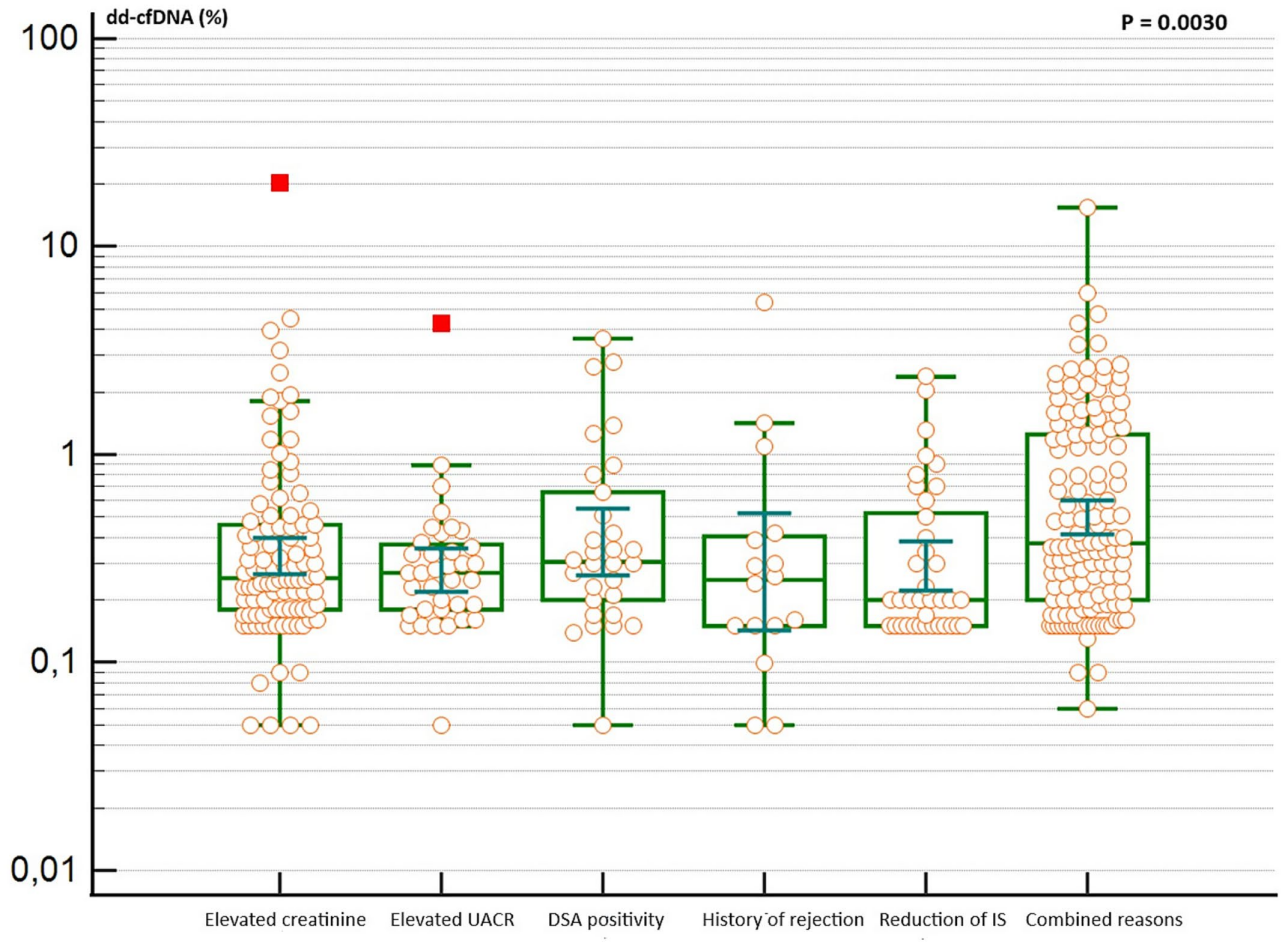
dd-cfDNA (%) – comparison of subgroups based on the reason for the measurement (log transformation). Distribution of donor-derived cell-free DNA (dd-cfDNA) expressed as percentage according to the primary clinical indication for the first dd-cfDNA measurement. Measurements were classified into six groups: elevated serum creatinine, elevated urinary albumin-to-creatinine ratio (UACR), donor-specific antibody (DSA) positivity, history of rejection, planned immunosuppression reduction, and combined indication (defined as the presence of ≥2 concurrent clinical indications). Box plots represent the median and interquartile range (IQR), whiskers indicate the range, and individual measurements are shown as circles. Overall group differences were assessed using the Kruskal–Wallis test.

**Figure 4:**
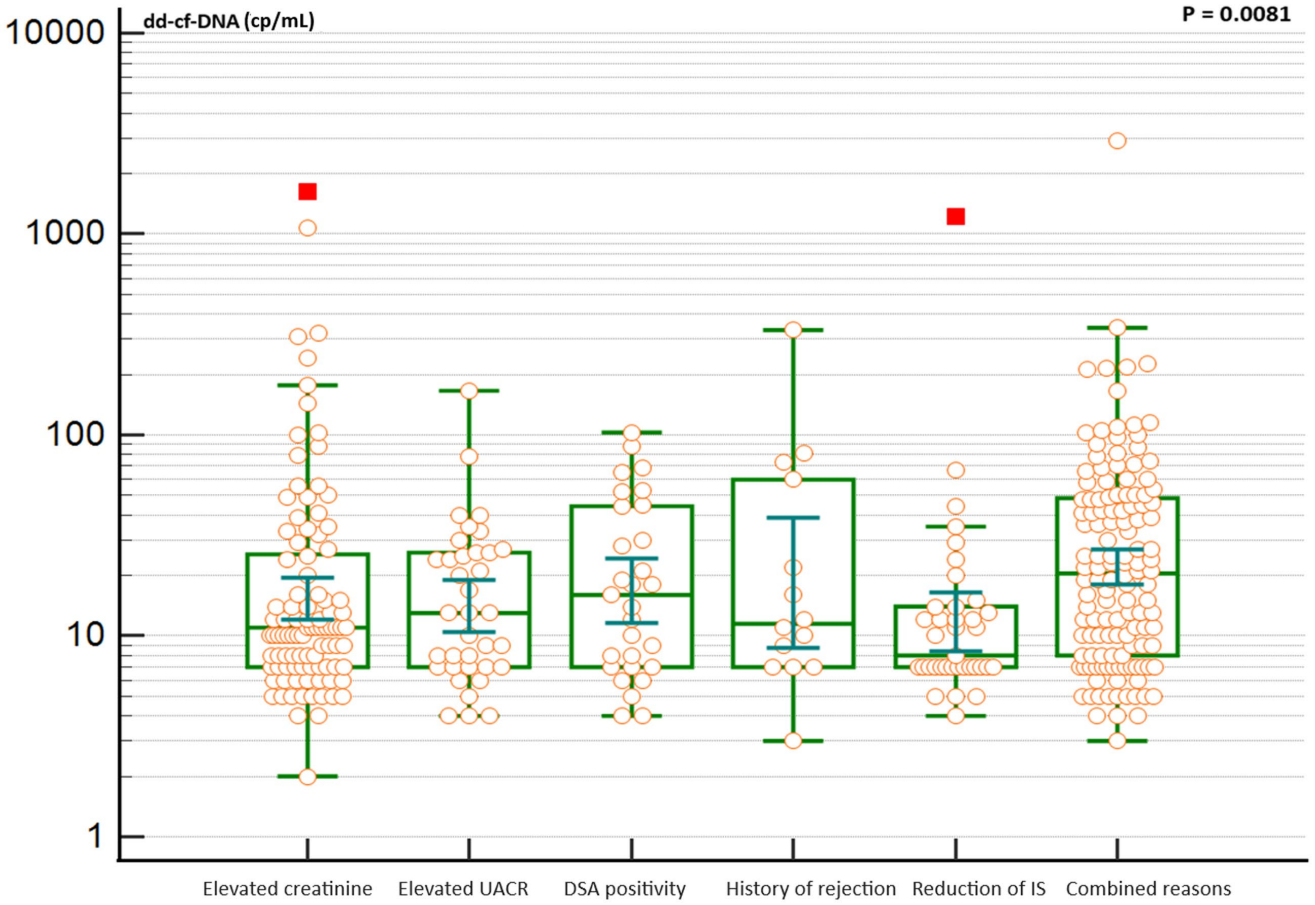
dd-cfDNA (cp/mL) – comparison of subgroups based on the reason for the measurement (log transformation). Distribution of donor-derived cell-free DNA (dd-cfDNA) expressed as absolute concentration (cp/mL plasma) according to the primary clinical indication for the first dd-cfDNA measurement. Measurements were classified into six groups: elevated serum creatinine, elevated urinary albumin-to-creatinine ratio (UACR), donor-specific antibody (DSA) positivity, history of rejection, planned immunosuppression reduction, and combined indication (defined as the presence of ≥2 concurrent clinical indications). Box plots represent the median and interquartile range (IQR), whiskers indicate the range, and individual measurements are shown as circles. Overall group differences were assessed using the Kruskal–Wallis test.

The dd-cfDNA distributions (percentages and copies/mL) across the subgroups are depicted in Figures 3, 4, respectively. Overall, the test results demonstrated large heterogeneity across groups, with values ranging from low to very high, particularly in Groups 1 and 6.

### Predictors of biopsy

In the multivariable logistic regression adjusting for age, sex, time post-transplant, baseline creatinine level, and baseline UACR value (Table 3), an elevated UACR value as the indication for testing emerged as the strongest independent predictor for biopsy (odds ratio [OR], 3.02; 95% confidence interval [CI], 1.39–6.58; *p* = 0.005).

**Table 3:** Multivariable logistic regression analysis of factors associated with kidney biopsy following dd-cfDNA measurement.

Outcome: biopsy	Odds ratio	95% CI	*p*-value
Total cfDNA (cp/mL)	1,0000	0.9999–1.0000	0.3081
dd-cfDNA (%)	1.2580	0.9498–1.6661	0.1094
dd-cfDNA (cp/mL)	0.9977	0.9931–1.0024	0.5328
Group 1 (elevated creatinine level)	1.0912	0.5205–2.2876	0.8172
Group 2 (elevated UACR value)	3.0234	1.3886–6.5829	**0.0053**
Group 3 (DSA positivity)	0.8451	0.3988–1.7909	0.6605
Group 4 (history of rejection)	0.9787	0.3976–2.4090	0.9626
Group 5 (planned IS reduction)	0.2737	0.0692–1.0824	0.0647
Group 6 (combined reason)	1.7630	0.5031–6,1777	0.3755
Follow-up measurements	1.0012	0.9974–1.0051	0.5328

DSA, donor-specific antibodies; dd-cfDNA, donor-derived cell-free DNA; UACR, urinary albumin-to-creatinine ratio; IS, immunosuppression.

Odds ratios (ORs) are presented with 95% confidence intervals (CIs). Multivariable logistic regression models were adjusted for age at kidney transplantation, sex, time from kidney transplantation to dd-cfDNA measurement, baseline serum creatinine, and baseline urinary albumin-to-creatinine ratio (UACR). Clinical indications for dd-cfDNA testing and follow-up measurements were entered as binary variables (yes/no). Bold values indicate statistically significant results (*p* < 0.05).

In multivariable logistic regression adjusted for age, sex, time post-transplant, baseline creatinine, and baseline UACR, elevated UACR as the testing indication was the only independent predictor of subsequent biopsy (OR 3.02, 95% CI 1.39–6.58; *p* = 0.005). Neither dd-cfDNA percentage nor copies/mL independently influenced biopsy decision making.

Multivariable linear regression showed that dd-cfDNA levels varied by reporting format and clinical context, with copies/mL being associated with elevated UACR, DSA positivity, history of rejection, and follow-up testing, whereas dd-cfDNA percentages were associated with elevated creatinine, elevated UACR, DSA positivity, and combined indications. ROC analysis demonstrated the limited discriminatory ability of dd-cfDNA for predicting biopsy, confirming that dd-cfDNA was not routinely used to guide biopsy decisions during the study period.

All detailed regression results, ROC analyses, and group characteristics for measurements following biopsy are provided in Supplementary Tables S4–S6 and Supplementary Figure S12.

### Biopsy-proven rejection versus no rejection

In this analysis, all biopsy-proven TCMR, ABMR, and mixed rejection cases were classified into a rejection group (*n* = 34; TCMR, 6; ABMR, 25; mixed, 3). Measurements with negative biopsy findings or other non-rejection diagnoses were categorized into the non-rejection group (*n* = 39; negative histology, 32; BKVAN, 2; interstitial fibrosis and tubular atrophy, 2; and recurrent disease, 3). Patients with borderline changes (*n* = 7) and measurements with non-representative or technically unreliable histology (*n* = 2) were excluded from the analysis.

Patients in the rejection group were significantly younger at transplantation (38 ± 17.1 vs. 48.6 ± 14.9, *p* = 0.0235), whereas sex distribution and time since transplantation were comparable. The two groups did not differ significantly in terms of baseline creatinine level, baseline UACR value, or clinical indications that prompted dd-cfDNA testing. In contrast, dd-cfDNA levels exhibited a clear separation between groups; median dd-cfDNA values were significantly higher in the rejection group, both when expressed as percentages (1.18% vs. 0.32%, *p* < 0.0001) and absolute copy numbers (36 vs. 11 copies/mL, *p* < 0.0001).

A comprehensive summary of all measurements followed by biopsies, including those excluded from the main analysis, is provided in Supplementary material. The summary encompasses Supplementary Figure S13 (dd-cfDNA measurements followed by biopsy and comparisons between non-rejection and rejection subgroups), Supplementary Figure S14 (biopsy results), Supplementary Figures S15–S17 (total cfDNA, dd-cfDNA [%], and dd-cfDNA [copies/mL] comparisons between non-rejection histology and rejection subgroups) and Supplementary Table S3 (group characteristics for measurements followed by biopsy).

#### Predictors of rejection

Multivariable logistic regression adjusted for age, sex, time post-transplant, baseline creatinine level, and baseline UACR identified dd-cfDNA percentage (OR, 5.46; 95% CI, 2.89–13.70; *p* = 0.0035) and dd-cfDNA copies/mL (OR, 7.05; 95% CI, 1.01–21.09; *p* = 0.0062) as independent predictors of biopsy-proven rejection (Table 4).

**Table 4:** Group characteristics: non-rejection versus rejection.

Patients: n = 53 Measurements: n = 73	Non-rejection Patients: *n* = 32 Measurements: *n* = 39	Rejection Patients: *n* = 21 Measurements: *n* = 34	*p*-value
Sex: male (%)	*n* = 28 (87.5%)	*n* = 16 (76.2%)	0.3163
Age at kidney transplantation (years) ± SD	48.6 ± 14.9 min, 22; max, 88	38 ± 17.1 min, 5; max, 69	**0.0235**
Time post-kidney transplantation when dd-cfDNA was performed (months), median (IQR, 25th–75th percentile)	Median, 58 (IQR, 10–153)	Median, 94 (IQR, 47–161)	0.3063
Total cfDNA (cp/mL), median (IQR, 25th–75th percentile)	Median, 4,014 (IQR, 2,820.25–6,832.25)	MEDIAN, 3,525 (IQR, 2,220–5,631)	0.1773
dd-cfDNA (%), median (IQR, 25th–75th percentile)	Median, 0.32 (IQR, 0.17–0.41)	Median, 1.18 (IQR, 0.51–1.86)	**<0.0001**
dd-cf-DNA (cp/mL), median (IQR, 25th–75th percentile)	Median, 11 (IQR, 7–26.5)	Median, 36 (IQR, 19–71)	**<0.0001**
Baseline creatinine level (mg/dL), median (IQR, 25th–75th percentile)	Median, 2.2 (IQR, 1.7–2.7)	Median, 1.8 (IQR, 1.5–2.36)	0.0984
Baseline UACR value (mg/g), median (IQR, 25th–75th percentile)	Median, 54.4 (IQR, 27.5–221)	Median, 107 (IQR, 26.4–322)	0.4978
Reasons for dd-cfDNA measurement			
Group 1 (elevated creatinine level)	*n* = 10 (25.6%)	*n* = 5 (14.7%)	0.4265
Transient increase	*n* = 3	*n* = 0	
Unsatisfactory graft function	*n* = 6	*n* = 2	
Group 2 (elevated UACR value)	*n* = 6 (15.4%)	*n* = 1 (2.9%)	0.2604
Group 3 (DSA positivity)	*n* = 1 (2.6%)	*n* = 3 (8.8%)	0.3325
Group 4 (history of rejection)	*n* = 3 (7.7%)	*n* = 0	0.2431
Group 5 (planned IS reduction)	*n* = 1 (2.6%)	*n* = 0	1.0000
Group 6 (combined reason)	*n* = 13 (33.3%)	*n* = 18 (52.9%)	0.5569
Follow-up measurements	*n* = 5 (12.8%)	*n* = 7 (20.6%)	0.7201
Single/multiple measurements	*n* = 31 (79.6%)/*n* = 8 (20.5%)	*n* = 21 (61.8%)/*n* = 13 (38.2%)	0.3366
Biopsy following dd-cfDNA (months after measurement), median (IQR, 25th–75th percentile)	median, 1 (IQR, 0–4)	median, 1 (IQR, 0–1)	0.3487

SD, standard deviation; IQR, interquartile range; DSA, donor-specific antibodies; dd-cfDNA, donor-derived cell-free DNA; UACR, urinary albumin-to-creatinine ratio; IS, immunosuppression.

Data are presented as mean ± standard deviation (SD), median (interquartile range [IQR]), or number (%), as appropriate. *p*-values were calculated using the chi-square or Fisher’s exact test for categorical variables, Student’s *t*-test for normally distributed continuous variables, and the Mann–Whitney U test for non-normally distributed continuous variables. Rejection was defined as biopsy-proven rejection. Bold values indicate statistically significant results (*p* < 0.05).

Among the clinical indications for testing, DSA positivity was the only variable independently associated with rejection (OR, 9.00; 95% CI, 1.62–14.89; *p* = 0.0009), consistent with previous reports (18).

The “planned IS reduction” variable (group 5) was excluded from the regression owing to zero events in the rejection group, causing complete separation due to the selection bias of this group. The variable “history of rejection” (group 4) was retained but showed no significant association (OR, 0.88; *p* = 0.3553). No other clinical indication was significantly associated with rejection (Table 5).

**Table 5:** Multivariable logistic regression analysis of factors associated with biopsy-proven rejection.

Outcome: rejection	Odds ratio	95% CI	*p*-value
Total cfDNA (cp/mL)	1.0000	0.9998–1.0001	0.7127
dd-cfDNA (%)	5.4647	2.8987–13.7052	**0.0035**
dd-cf-DNA (cp/mL)	7.0520	1.0145–21.0908	**0.0062**
Group 1 (elevated creatinine level)	0.4351	0.1214–1.5587	0.0808
Group 2 (elevated UACR value)	2.8668	0.7684–5.2080	0.1557
Group 3 (DSA positivity)	9.0067	1.6251–14.8992	**0.0009**
Group 4 (history of rejection)	0.8840	0.1654–4.7243	0.3553
Group 6 (combined reason)	1.0175	0.8025–1.2900	0.8863
Follow-up measurements	1.7630	0.5031–6.1777	0.3755

DSA, donor-specific antibodies; dd-cfDNA, donor-derived cell-free DNA; UACR, urinary albumin-to-creatinine ratio; IS, immunosuppression.

Odds ratios (ORs) are presented with 95% confidence intervals (CIs). Multivariable logistic regression models were adjusted for age at kidney transplantation, sex, time from kidney transplantation to dd-cfDNA measurement, baseline serum creatinine, and baseline urinary albumin-to-creatinine ratio (UACR). Clinical indications for dd-cfDNA testing and follow-up measurements were entered as binary variables (yes/no). Group 5 (planned immunosuppression reduction) was excluded from the regression model because no rejection events occurred in this subgroup, resulting in complete separation. Bold values indicate statistically significant results (*p* < 0.05).

**Figure 5:**
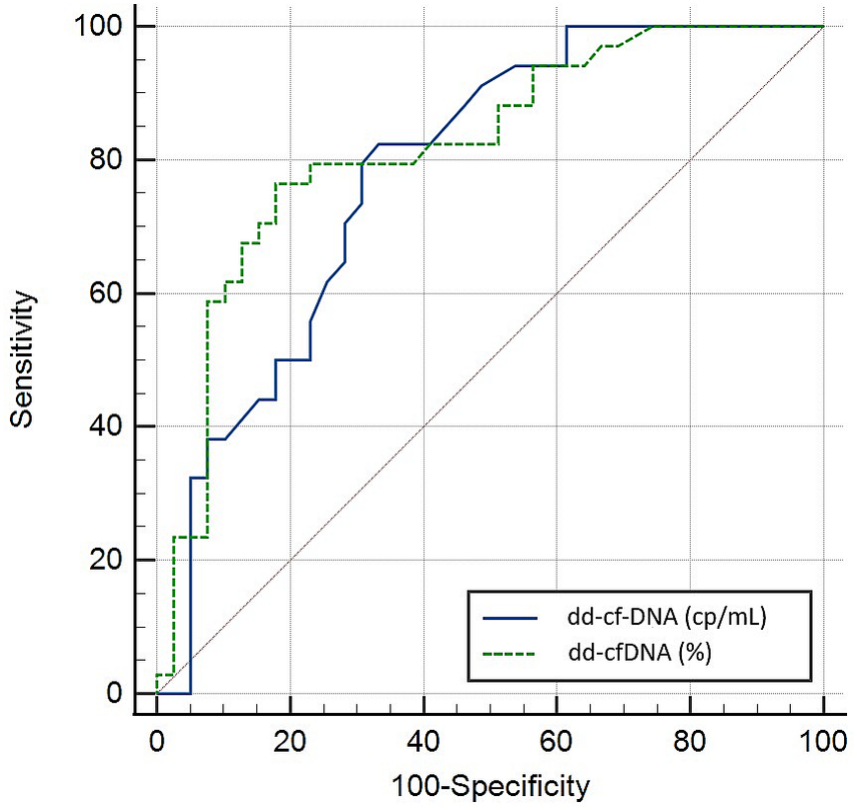
Comparative ROC analysis of dd-cfDNA (cp/mL and %) for the prediction of rejection. Receiver operating characteristic (ROC) curves comparing the diagnostic performance of donor-derived cell-free DNA (dd-cfDNA) expressed as absolute concentration (cp/mL plasma) and percentage (%) for predicting biopsy-proven rejection among dd-cfDNA measurements followed by a clinically indicated kidney biopsy.dd-cfDNA (copies/mL): optimal cut-off >16 copies/mL; sensitivity 82.4%; specificity 66.7%; AUC 0.781; p < 0.001.dd-cfDNA (%): optimal cut-off >0.49%; sensitivity 76.5%; specificity 82.1%; AUC 0.820; *p* < 0.001. There was no significant difference between the AUCs of the two reporting formats (DeLong test, *p* = 0.3642).

**Figure 6:**
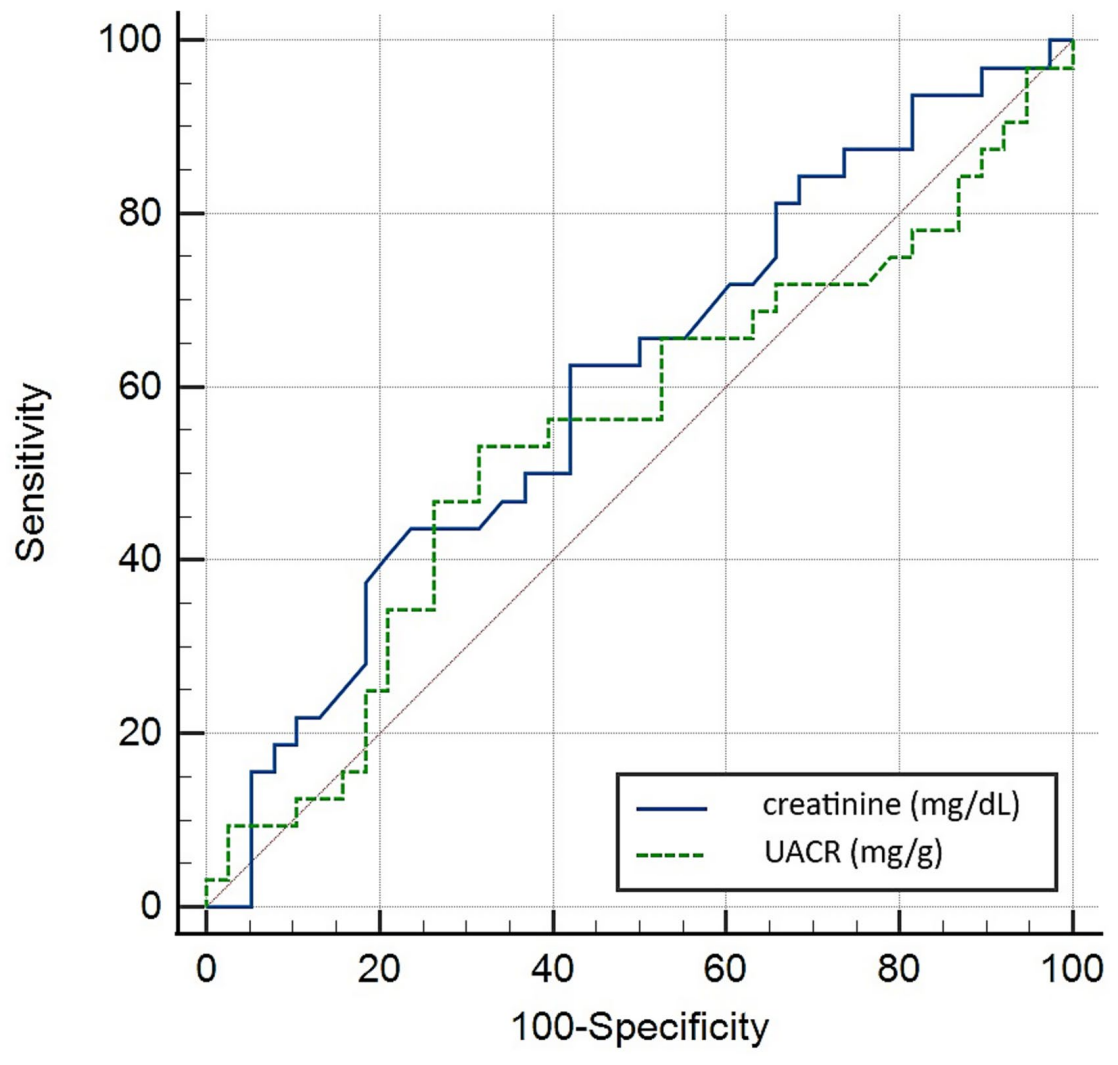
Comparative ROC analysis of creatinine base line (mg/dL) and UACR baseline (mg/g) for the prediction of rejection. Receiver operating characteristic (ROC) curves comparing the diagnostic performance of baseline serum creatinine (mg/dL) and baseline urinary albumin-to-creatinine ratio (UACR; mg/g) for predicting biopsy-proven rejection among dd-cfDNA measurements followed by a clinically indicated kidney biopsy. Baseline serum creatinine (mg/dL): optimal cutoff >1.88 mg/dL; sensitivity 62.5%; specificity 59.0%; AUC 0.615; *p* = 0.090. Baseline UACR (mg/g): optimal cut-off >85.5 mg/g; sensitivity 53.1%; specificity 68.4%; AUC 0.547; *p* = 0.509.

ROC curve analysis demonstrated good discriminatory performance of dd-cfDNA for biopsy-proven rejection among measurements followed by a clinically indicated biopsy. For dd-cfDNA expressed as copies/mL, the optimal cut-off (>16 copies/mL; Youden index J = 0.4902) yielded 82.4% sensitivity and 66.7% specificity (AUC, 0.781; *p* < 0.001). For dd-cfDNA expressed as percentage, the optimal threshold (>0.49%; Youden index J = 0.5852) yielded 76.5% sensitivity and 82.1% specificity (AUC, 0.820; p < 0.001). The diagnostic performance of the two reporting formats did not differ significantly (difference between AUCs = 0.0385; 95% CI, −0.0446 to 0.122; *p* = 0.3642, DeLong test; Figure 5). In contrast, baseline serum creatinine and UACR demonstrated only limited discriminatory ability for rejection, with AUCs of 0.615 (criterion >1.88 mg/dL; sensitivity 62.5%; specificity 59%; *p* = 0.090) and 0.547 (criterion >85.5 mg/g; sensitivity 53.1%; specificity 68.4%; *p* = 0.509), respectively (Figure 6).

To further explore the potential clinical utility of dd-cfDNA for identifying patients at low rejection risk, we investigated dd-cfDNA levels across 32 measurements with completely negative biopsy histology. Using the combined ROC-derived thresholds (>16 copies/mL and >0.49%), 21 of 32 measurements (66%) exhibited dd-cfDNA values below both cut-off values. In contrast, only 5 of 34 measurements (14.7%) in the rejection group fell below both the thresholds. Thus, low dd-cfDNA correctly classified approximately two-thirds of negative biopsies, whereas only a small proportion of rejection cases fell below both thresholds. These findings suggest that low dd-cfDNA may help identify recipients with a lower probability of rejection, although this observation requires prospective validation.

**Figure 7:**
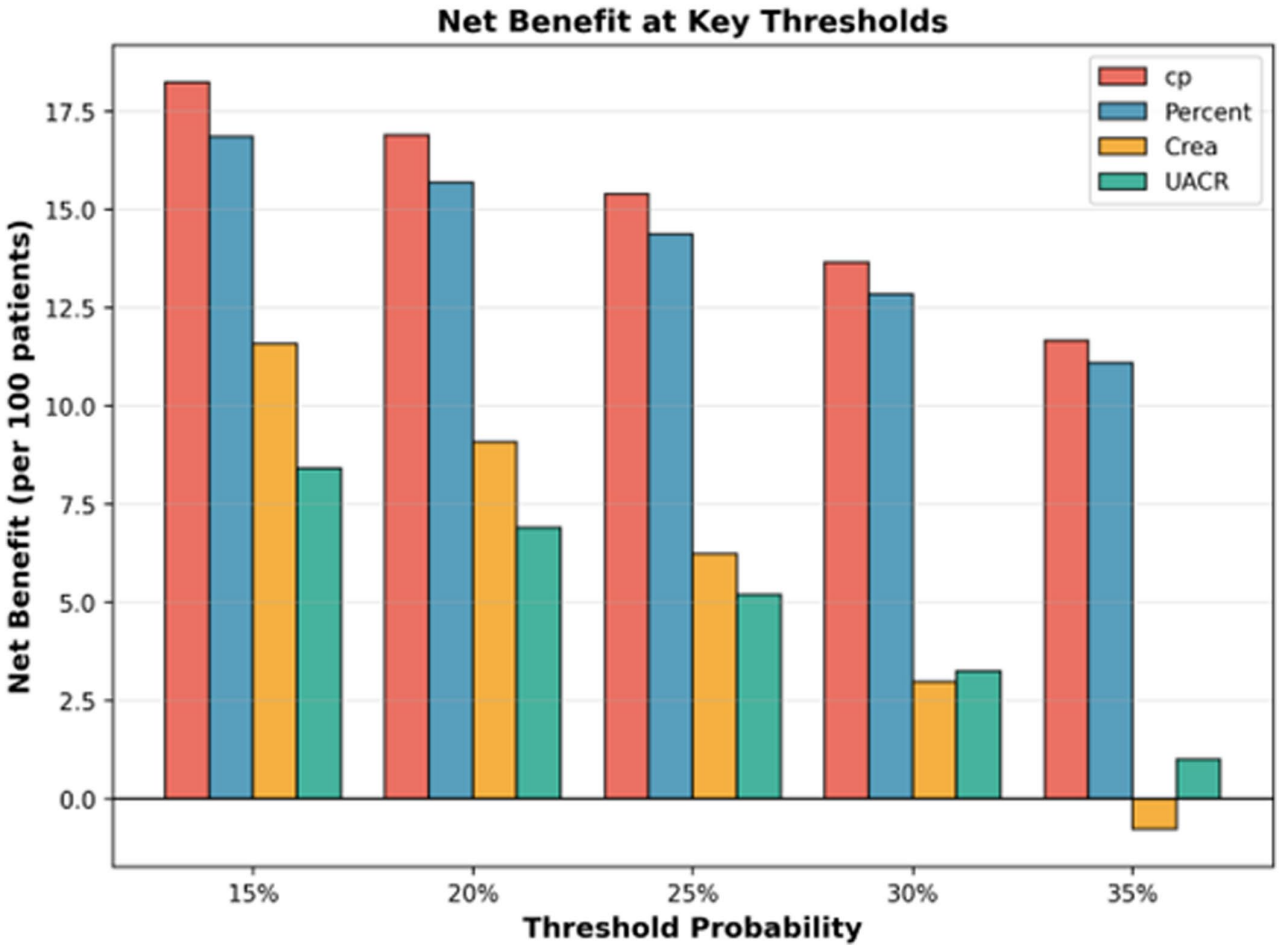
Decision curve analysis. Decision curve analysis (DCA) demonstrating the net benefit of donor-derived cell-free DNA (dd-cfDNA) expressed as absolute concentration (copies/mL plasma) and percentage (%), compared with baseline serum creatinine and baseline urinary albumin-to-creatinine ratio (UACR), for predicting biopsy-proven rejection. Bars represent the standardized net benefit per 100 patients across clinically relevant threshold probabilities (15, 20, 25, 30, and 35%). At all evaluated thresholds, both dd-cfDNA reporting formats demonstrated a greater clinical net benefit than baseline serum creatinine and UACR, supporting the incremental value of dd-cfDNA in guiding biopsy decisions.

To complement these analyses, we performed a DCA comparing dd-cfDNA (% and copies/mL) with baseline creatinine levels and UACR values. Across clinically relevant threshold probabilities, dd-cfDNA offered a considerably greater net benefit than either creatinine or UACR, two parameters that deeply impacted biopsy decisions in routine practice (Figure 7). This finding indicates that dd-cfDNA can significantly enhance decision-making when integrated with established clinical markers.

## Discussion

We hypothesized that, in real-world clinical practice, dd-cfDNA testing is primarily initiated based on traditional markers of graft injury, such as serum creatinine and albuminuria, rather than on dd-cfDNA-driven decision-making itself, while dd-cfDNA would nevertheless provide superior independent discrimination of biopsy-proven rejection. Our results confirm this hypothesis. Although elevated UACR emerged as the only independent predictor of biopsy performance, both dd-cfDNA percentage and absolute copy number independently predicted biopsy-proven rejection and outperformed baseline creatinine and UACR in diagnostic accuracy.

Across clinical scenarios, dd-cfDNA values differed significantly and demonstrated distinct patterns depending on the reporting format. Copies/mL were higher in measurements prompted by elevated UACR values, DSA positivity, history of rejection, and follow-up testing, whereas percentages increased with elevated creatinine levels, elevated UACR values, DSA positivity, and combined reasons. These results suggest that the two reporting formats can partially capture the distinct biological dimensions of graft injury, with copies/mL more closely reflecting active alloimmune damage and percentages being more sensitive to shifts in graft function and perfusion (3–5, 7, 19–22). However, this interpretation is speculative because of the small sample size of each group.

This study provides several practical insights from the literature. First, it systematically characterized real-world test utilization across six predefined clinical indications, providing a structured overview of how dd-cfDNA is requested in routine practice. Second, it demonstrated strong associations between dd-cfDNA levels and established clinical and immunological risk markers, highlighting its value as an adjunctive indicator of graft injury. Notably, baseline creatinine level, baseline UACR value, and clinical indications for testing did not substantially discriminate between the rejection and non-rejection groups despite obvious differences in dd-cfDNA levels between these groups. This finding emphasizes that routine clinical parameters alone were inadequate to distinguish alloimmune injury in this cohort, whereas dd-cfDNA effectively discriminated between histological outcomes. Lastly, despite these associations, biopsy decisions continued to depend predominantly on conventional clinical parameters, including creatinine and proteinuria, indicating the early stage of dd-cfDNA implementation during the study period.

This pattern reflects the implementation-science literature highlighting the variability in integrating molecular biomarkers into clinical workflows (23). Current guidelines from the American Society of Transplant Surgeons underscore dd-cfDNA as a decision-support tool that complements established clinical and histological evaluations, rather than replacing it (24). In several centers, particularly in the United States, low dd-cfDNA values increasingly inform decisions to defer biopsy when other findings are not concerning. However, this approach is not yet universal. Although dd-cfDNA has demonstrated value in for-cause biopsies, its ability to detect rejection in surveillance biopsies has been more variable; in one cohort, elevated dd-cfDNA predicted rejection in clinically indicated biopsies, but not in scheduled surveillance biopsies (25).

Collectively, these findings emphasize two key points in clinical practice. First, UACR remains a strong trigger for biopsy in real-world practice; however, its specificity for alloimmune injury remains limited, particularly in non-alloimmune conditions, including inflammatory, infectious, or hemodynamic processes, where proteinuria unreliably reflects alloimmune injury (11, 13, 19). Second, dd-cfDNA offers high-value complementary information with considerably better specificity for rejection than creatinine or UACR (26, 27). Our findings support the hypothesis that combining standard clinical markers with dd-cfDNA may improve risk stratification and biopsy decision-making in kidney transplant recipients. However, the present study was not designed to formally evaluate the incremental value of dd-cfDNA across specific clinical scenarios, and this concept should be confirmed in prospective multicenter studies. This key point aligns with the large and convincing body of evidence demonstrating the substantial diagnostic value of dd-cfDNA in for-cause biopsy contexts (3, 7, 8, 13, 20–22, 28).

This study has several limitations. This was a single-center retrospective analysis in which dd-cfDNA testing and biopsy decisions were clinician-directed rather than protocolized, which may limit generalizability. Approximately half of all dd-cfDNA measurements were excluded, largely because of protocol-mandated testing in interventional ABMR trials or missing key data; therefore, non-random missingness and selection bias cannot be excluded. Because some recipients contributed multiple measurements, residual within-patient correlation was possible despite sensitivity analyses being restricted to the first test per patient. Diagnostic performance metrics were derived only in patients who underwent clinically indicated biopsy and therefore reflect test performance in a biopsy-selected, higher-risk population; partial verification (work-up) bias is possible, and the reported cut-offs should not be extrapolated to clinically stable, unbiopsied recipients. Although the overall cohort comprised 571 dd-cfDNA measurements, only 82 measurements were followed by biopsy, including 34 biopsy-proven rejection events. Consequently, the study was not sufficiently powered to perform robust stratified or interaction analyses evaluating the incremental value of dd-cfDNA across individual clinical testing indications (e.g., elevated UACR, DSA positivity, or elevated creatinine). Future prospective multicenter studies with larger numbers of biopsy-confirmed events are needed to determine the incremental value of dd-cfDNA within specific clinical scenarios and to evaluate whether combining clinical indications with dd-cfDNA improves risk stratification and biopsy decision-making. In addition, urinary albumin-to-creatinine ratio (UACR) was the standard measure of albuminuria routinely used for decision making in our center, whereas urine protein-to-creatinine ratio (UPCR) and 24-h urinary protein excretion were not always available for a systematic analysis in this retrospective cohort. Future studies comparing different measures of proteinuria may help determine which parameter is most informative for outcome and/or guiding diagnostic measures such as biopsy or dd-cfDNA testing. Borderline rejection biopsies were excluded from rejection versus non-rejection analyses, which may further limit the generalizability and preclude conclusions regarding dd-cfDNA performance in borderline changes. In addition, dd-cfDNA may increase in non-alloimmune forms of graft injury, including BK viremia (with or without nephropathy), infection, ischemia–reperfusion injury, or acute tubular injury; therefore, dd-cfDNA should be interpreted alongside creatinine, proteinuria, DSA, and clinical context. Finally, the rejection cohort was enriched for ABMR, limiting the power to evaluate isolated TCMR, and the long-term graft outcomes were not assessed. This study has several strengths. It represents one of the larger single-center real-world cohorts evaluating dd-cfDNA in routine post-kidney-transplant care, with a multiyear observation period that spans different stages of clinical familiarity and implementation of the test. A detailed chart review allowed the granular classification of testing indications and clinical intent rather than relying on administrative coding. The availability of paired dd-cfDNA percentage and absolute copy number values enabled the comparison of these two reporting formats in the same clinical scenarios, an area that remains relatively underexplored (29, 30). The simultaneous availability of biopsy findings in a substantial proportion of cases further allowed correlation of dd-cfDNA with histology and multivariable analyses adjusted for key clinical confounders including kidney function and albuminuria. Together, these features provide a comprehensive description of the use of dd-cfDNA in everyday clinical practice.

## Conclusion

In conclusion, dd-cfDNA showed strong discriminatory ability for biopsy-proven rejection and reliably reflected the underlying graft injury across diverse clinical contexts, particularly in patients with proteinuria, DSA positivity, or a history of rejection. Although dd-cfDNA was not routinely integrated into biopsy decision-making during the study period, its independent association with rejection and promising diagnostic performance support its role as a complementary biomarker for clinical assessment and biopsy guidance.

These findings reinforce the existing evidence indicating the value of dd-cfDNA in for-cause evaluations and support its integration into multimodal monitoring strategies that incorporate laboratory, immunological, and functional parameters for a more refined and noninvasive kidney allograft health assessment. To define the optimal implementation of dd-cfDNA in routine care and clarify how it may contribute to more precise and tailored biopsy utilization after kidney transplantation, prospective studies are warranted.

## Data Availability

The original contributions presented in the study are included in the article/Supplementary material, further inquiries can be directed to the corresponding author.
