# Supplementary material for: Real-world utilization patterns and association of dd-cfDNA with biopsy and rejection
Source: Front Med (Lausanne). 2026 Jul 22;13:1890561. doi: 10.3389/fmed.2026.1890561 (PMC13439412; doi:10.3389/fmed.2026.1890561)
Supplement: Supplementary file 1 [file Supplementary_file_1.docx]

**Supplementary Material for the manuscript:**
*Clinical indications for donor-derived cell-free DNA and their association with biopsy and biopsy-proven rejection in kidney transplantation*

**List of Supplementary Tables and Figures**

**1. Supplementary Methods:**

1A. Detailed method description for donor-derived cell-free DNA quantification

1B. Statistical analysis

**2. Supplementary Tables**

**Table S1** Baseline characteristics of patients with included versus excluded dd-cfDNA measurements

**Table S2** Basic characteristics – single vs. multiple measurements

**Table S3** Group characteristics – KTx ≤ 12 months, 12–60 months, > 60 months

**Table S4** Group characteristics – measurements followed by biopsy

**Table S5** Multivariable linear regression analysis of factors associated with dd-cfDNA (cp/mL)

**Table S6** Multivariable linear regression analysis of factors associated with dd-cfDNA (%)

**3. Supplementary Figures**

**Figure S1** Reasons for dd-cfDNA measurements – total (whole group)

**Figure S2** Types of rejection for “history of rejection” reason of dd-cfDNA measurement

**Figure S3** Reasons for dd-cfDNA measurements – only reasons (single vs. multiple measurements)

**Figure S4** Total dd-cfDNA – comparison of subgroups based on the reason for the measurement

**Figure S5–S7** Total dd-cfDNA, dd-cfDNA (%), dd-cfDNA (cp/mL) – comparison of multiple vs. single measurement group (log transformation)

**Figure S8–S10** Total dd-cfDNA, dd-cfDNA (%), dd-cfDNA (cp/mL) – comparison of post-transplantation time groups (KTx ≤ 12 m, 12–60 m, > 60 m)

**Figure S11** Reasons for dd-cfDNA measurements – KTx ≤ 12 months, KTx 12-60 months, KTx > 60 months

**Figure S12** Comparative ROC analysis of dd-cfDNA (cp/mL and %) for the prediction of biopsy

**Figure S13** dd-cfDNA measurements followed by biopsy and comparisons between non-rejection histology vs. rejection subgroups

**Figure S14** Biopsy results

**Figure S15-17** Total dd-cfDNA, dd-cfDNA (%), dd-cfDNA (cp/mL) – groups comparison of non-rejection histology and rejection subgroups

**1. Supplementary Methods:**

1A. Material and Methods:

**Study design and setting**

This was a systematic retrospective observational study conducted at the Kidney Transplant Center of Charité–Universitätsmedizin Berlin beginning with the introduction of donor-derived cell-free DNA (dd-cfDNA) testing in 2020. In this study, “real-world” refers to the use of dd-cfDNA as part of routine clinical care, with clinician-directed, non-protocol test ordering and interpretation, rather than testing mandated by a research protocol or fixed biopsy algorithm.

The objectives were to:

1. identify and characterize the clinical indications for dd-cfDNA testing,
2. evaluate testing patterns over time,
3. determine which patient characteristics and dd-cfDNA results were associated with subsequent biopsy.

**Study population and exclusions**

Between 29 April 2020 and 15 May 2025, 1,079 dd-cfDNA measurements were performed in kidney transplant recipients.

The following were excluded:

- multiorgan transplant recipients (kidney–pancreas, kidney–liver, pancreas alone)
- patients enrolled in interventional clinical trials with protocol-mandated dd-cfDNA testing (patients excluded because of participation in interventional studies predominantly had active or previous ABMR, reflecting trial inclusion criteria. As expected, these patients had higher rates of DSA positivity, worse graft function, and higher dd-cfDNA values than the routine-care cohort)
- measurements with missing key clinical context required to classify indication or adjudicate outcomes (e.g., unavailable donor-specific antibody [DSA] status, insufficient documentation, unclear indication)

Interventional-trial cases were excluded because test indications and biopsy decisions in this setting were protocol-driven and therefore did not reflect routine clinician-directed practice. Exclusions due to missing information reflected absence of essential clinical data rather than biological test performance.

After exclusions, 571 dd-cfDNA measurements from 313 kidney-only recipients were available for analysis (Figure 1).

**Repeated measurements**

Recipients were categorized as having:

- a single dd-cfDNA measurement
- multiple dd-cfDNA measurements

For patients with multiple measurements:

- only the first measurement was used to determine indication
- subsequent measurements were classified as
  - follow-up: ≤6 months since prior measurement
  - new diagnostic test: >6 months since prior measurement

For each dd-cfDNA measurement, it was recorded whether the test was followed by a diagnostic kidney allograft biopsy.

**Baseline laboratory values**

For each dd-cfDNA measurement:

- baseline serum creatinine
- baseline urine albumin-to-creatinine ratio (UACR)

were defined as the mean of 3–5 values prior to the index test. These 3–5 measurements were typically obtained within the preceding 3–6 months, reflecting routine outpatient follow-up intervals. Laboratory values from the day prompting dd-cfDNA ordering were not included in baseline calculations.

**Ascertainment and classification of testing indications**

Indications were not prospectively coded in the order entry system. They were therefore reconstructed retrospectively through structured review of the electronic medical record, including:

- dd-cfDNA test order details
- contemporaneous clinic and inpatient notes
- laboratory results
- clinician correspondence

Classification followed prespecified decision rules and was discussed within the study team in consensus meetings. Ambiguous cases were adjudicated by consensus. When the primary indication could not be determined with sufficient confidence, the case was labeled “uncertain” and excluded from indication-specific analyses.

The reasons for the examination indication were subsequently assessed as follows:

1. **Group 1:** elevated creatinine level (>50% above baseline)

- Subgroup A: transient creatinine increase, defined as a return to the baseline level within the subsequent three creatinine measurements following the dd-cfDNA test
- Subgroup B: unsatisfactory graft function, defined as a persistently elevated creatinine level without other laboratory or clinical reasons,

1. **Group 2:** increased UACR value (persistently >50% above baseline),
2. **Group 3:** DSA positivity,
3. **Group 4:** history of rejection (the rejection type was also recorded in this case),
4. **Group 5:** planned immunosuppression (IS) reduction for any reason,
5. **Group 6:** combined reasons (elevated creatinine level, increased UACR value +/- DSA positivity),
6. **Follow-up measurements** (defined as subsequent measurements taken within 6 months from the previous measurement in the same patient) (Figure 1).

The indications for dd-cfDNA testing and the total number of measurements for each study group are presented in Supplementary Figure S1.

**Biopsy practice and availability of dd-cfDNA results**

During the study period, no protocol (time-driven) biopsies were performed; all kidney allograft biopsies were clinically indicated. dd-cfDNA results were generally available to treating clinicians at the time biopsy decisions were made. dd-cfDNA testing was not embedded in a mandatory biopsy algorithm, and no fixed dd-cfDNA threshold mandated or precluded biopsy. Decisions to biopsy reflected overall clinical judgment, including kidney function trajectory, proteinuria, immunologic risk (including DSA status), dd-cfDNA values, and the broader clinical context. Clinical familiarity with dd-cfDNA increased over time, which may have influenced test utilization and decision-making.

For histology-based subgroup analyses, all biopsy-proven rejection cases, including T cell–mediated rejection (TCMR), antibody-mediated rejection (ABMR), and mixed rejection, were classified as rejection. Biopsies exhibiting borderline changes were excluded from rejection versus non-rejection analyses. All other biopsy diagnoses, including recurrent or de novo glomerular disease, BK virus–associated nephropathy (BKVAN), tubular injury, or chronic interstitial fibrosis, were retained in the non-rejection group.

**dd-cfDNA quantification and histological classification**

dd-cfDNA and total cfDNA were measured using a single-nucleotide polymorphism (SNP)–based droplet digital PCR (ddPCR) assay. Results were reported as:

- relative fractions (%)
- absolute copy number (copies/mL)

For histopathological evaluation:

- T cell–mediated rejection (TCMR)
- antibody-mediated rejection (ABMR)
- mixed rejection

were classified as rejection. Borderline changes were excluded; all remaining diagnoses were classified as non-rejection.

**dd-cfDNA measurement**

For each patient, four informative single nucleotide polymorphisms (SNPs)-defined as SNPs for which the recipient was homozygous and the donor carried at least one heterozygous allele-were selected from a predefined panel of 45 SNPs. These four SNPs were used to quantify donor-derived cell-free DNA (dd-cfDNA) as follows:

dd-cfDNA (%) = donor alleles / (donor alleles + recipient alleles)

Results for SNPs with heterozygous donor genotypes were corrected by a factor of 2.

Total cell-free DNA (cfDNA) was extracted from up to 8 mL of plasma, and its concentration was determined as haploid genome copies using droplet digital PCR (ddPCR). Measured cfDNA concentrations were corrected for extraction efficiency and cfDNA fragmentation, as previously described [1].

The absolute concentration of dd-cfDNA (copies/mL plasma) was calculated by multiplying total cfDNA (copies/mL) by dd-cfDNA (%). The assay methodology, including donor-recipient discrimination based on informative SNPs, analytical validation, assay performance, and quality-control procedures, has been described previously Reineke M, et al. [1].

**Diagnostic performance analyses and verification bias**

Diagnostic performance analyses (AUC, thresholds, sensitivity/specificity, and decision-curve analysis) were conducted in the subset of dd-cfDNA measurements followed by biopsy. Because biopsies were performed selectively based on clinical judgment, the biopsied subset represents a higher-risk population, and these metrics describe test performance conditional on biopsy.

1. Reineke M, Loi L, Schröter I, et al..[High Concordance between ddPCR- and NGS-Based Quantification of Donor-Derived Cell-Free DNA Percentage after Kidney Transplantation.](https://pubmed.ncbi.nlm.nih.gov/42271643/) Clin Chem. 2026 Jun 11:hvag056. doi: 10.1093/clinchem/hvag056. Online ahead of print. PMID: 42271643

1B. Statistical analysis:

All statistical analyses were performed using MedCalc version 23.3.7 (member of the [International Association of Statistical Computing](http://www.iasc-isi.org/), Ostend, Belgium), a certified statistical program.

The characteristics of the study population were summarized using descriptive statistics (means, medians, standard deviations, IQRs, frequencies, and percentages). Using the Kolmogorov–Smirnov test, continuous variables were assessed for normality. Variables with a normal distribution were expressed as means ± standard deviations and compared between groups using Student’s t-test. Non-normally distributed variables were presented as medians and IQRs (25th–75th percentile) and compared using the Mann–Whitney U test. Categorical variables were compared using the chi‑square test or Fisher’s exact test, as appropriate.

To compare variables across multiple groups, analysis of variance (for age during kidney transplantation) and Kruskal–Wallis tests (for dd‑cfDNA metrics, creatinine, UACR, time from dd‑cfDNA measurement to biopsy, and reasons for measurement) were used.

Receiver operating curve (ROC) curve analysis was employed for evaluating the diagnostic accuracy of dd-cfDNA levels for predicting biopsy in the entire cohort and for predicting rejection in the subset of measurements followed by clinically indicatied biopsy. Optimal cut-off values were determined by maximizing the Youden index (J = sensitivity + specificity − 1). Because biopsies were performed selectively and not at random, ROC curves and cut-offs for rejection reflect test performance conditional on biopsy and may be affected by partial verification (work-up) bias. The DeLong test was used to compare the areas under the curves (AUCs) for dd-cfDNA expressed as percentage and as copies/mL.

To evaluate the associations between reasons for measurement and biopsy, and between reasons for measurement and biopsy‑proven rejection, logistic regression with adjustment for age at transplantation, sex, time post-transplantation when dd‑cfDNA testing was performed, baseline creatinine level, and baseline UACR value was used. Because only 34 rejection events were observed, these multivariable models may be at risk of over-parameterization and potential overfitting; therefore, results are interpreted as exploratory. dd-cfDNA percentage and copies/mL were highly correlated.

To evaluate the associations between dd‑cfDNA (copies/mL) and dd‑cfDNA (%) and reasons for measurement, multivariable linear regression with the same adjustments (age at transplantation, sex, time post-transplantation when dd‑cfDNA was performed, baseline creatinine level, and baseline UACR value) was employed. Because both dd-cfDNA (%) and dd-cfDNA (copies/mL) exhibited a right-skewed distribution, both variables were log-transformed before linear regression analyses. Clinical testing indications were entered into the models as binary dummy variables (yes/no), with the absence of the respective indication serving as the reference category. P-values < 0.05 were considered statistically significant. Multicollinearity was assessed using variance inflation factors (VIFs). No evidence of problematic multicollinearity was observed (all VIFs < 2)

Decision curve analysis (DCA) was performed for evaluating the clinical net benefit of dd-cfDNA (expressed as percentage and copies/mL) compared with baseline creatinine level and UACR value for predicting biopsy-proven rejection. Standardized net benefit was computed across various clinically meaningful threshold probabilities. The analysis followed the recommendations of Vickers and Elkin, with “treat-all” and “treat-none” strategies included as reference curves.

**2. Supplementary Tables**

**Table S1** Baseline characteristics of patients with included versus excluded dd-cfDNA measurements

|  | **Included**  **N = 313**  **measurements: n = 571** | **Excluded**  **N = 105**  **measurements: n = 508** | **P value** |
| --- | --- | --- | --- |
| **Sex: male (%)** | N = 209 (66.8%) | N = 74 (70.5) | 0.4834 |
| **Age at kidney transplantation (years) ± SD** | 42.9 ± 15.3  median, 43 | 42.6 ± 14.4  median, 45.8 | 0.8601 |
| **Total cfDNA (cp/mL), median (IQR, 25th–75th percentile)** | 4.307 (IQR, 2,726.5–8,254.5) | 3764 (IQR 2152-2609) | 0.6652 |
| **dd-cfDNA (%), median (IQR, 25th–75th percentile)** | 0.35 (IQR, 0.2–0.85) | 0.6 (IQR 0.3-1.1) | <0.0001 |
| **dd-cf-DNA (cp/mL), median (IQR, 25th–75th percentile)** | 16 (IQR, 8–44) | 26 (IQR 14-51) | <0.0001 |
| **Baseline creatinine (mg/dL), median (IQR, 25th–75th percentile)** | 1.88 (IQR, 1.53–2.44) | 1.56 (IQR 1.3-1.9) | <0.0001 |
| **Baseline UACR (mg/g), median (IQR, 25th–75th percentile)** | 79 (IQR, 27.3–324.5) | 87 (IQR 19.4-411.2) | 0.4541 |

dd-cfDNA, donor-derived cell-free DNA; cfDNA, cell-free DNA; DSA, donor-specific antibody; UACR, urine albumin-to-creatinine ratio; ABMR, antibody-mediated rejection; IQR, interquartile range.

Included measurements represent clinician-directed dd-cfDNA testing performed as part of routine clinical care. Excluded measurements predominantly originated from protocol-mandated testing within interventional studies (most commonly ABMR-focused trials) or from multiorgan transplant recipients, and therefore were not considered representative of real-world test utilization. Continuous variables are presented as mean ± SD or median (interquartile range) as appropriate; categorical variables are presented as number (percentage). Baseline serum creatinine and UACR were calculated as the mean of 3–5 measurements preceding the index dd-cfDNA test. “Included” refers to measurements retained in the primary analysis cohort; “Excluded” refers to multiorgan transplants, protocol-mandated study measurements, or cases with missing key clinical data. Differences between included and excluded cohorts were assessed using appropriate parametric or nonparametric tests; P values are provided for descriptive purposes and were not adjusted for multiple comparisons.

**Table S2** Basic characteristics – single vs. multiple measurements

| **patients: n = 313**  **measurements: n = 571** | **single measurement**  **n = 201 measurements**  **n = 201 patients** | **multiple measurements**  **n = 370 measurements**  **n = 112 patients** | **P value** |
| --- | --- | --- | --- |
| **sex – male (%)** | n = 141 (70,1 %) | n = 68 (60,7 %) | 0,1766 |
| **age at time of kidney transplantation (years) ± SD** | 42,8 ± 15,7  median 44  min 5, max 81 | 43,5 ± 14,6  median 41  min 11, max 84 | 0,4005 |
| **time after kidney transplantation when dd-cfDNA performed (months), median, [IQR 25th–75th percentile]** | median 86  IQR 26-154 | median 68  IQR 18-137 | 0,1370 |
| **total cfDNA (cp/mL), median, [IQR 25th–75th percentile]** | median 4303  IQR 2826-7603 | median 4307,5  IQR 2708-9083 | 0,4157 |
| **dd-cfDNA (%), median, [IQR 25th–75th percentile]** | median 0,26  IQR 0,17-0,45 | median 0,42  IQR 0,24-1,1 | **<0,0001** |
| **dd-cf-DNA (cp/mL), median, [IQR 25th–75th percentile]** | median 11  IQR 7-24 | median 23  IQR 10-58 | **<0,0001** |
| **creatinine base line (mg/dL), median, [IQR 25th–75th percentile]** | median 1,7  IQR 1,38-2,25 | median 1,98  IQR 1,6-2,5 | **<0,0001** |
| **UACR base line (mg/g), median, [IQR 25th–75th percentile]** | median 60  IQR 24-273 | median 100  IQR 31,5-331 | **0,0191** |
| **reasons for dd-cfDNA measurement** | | | |
| **group 1 (elevated creatinine) in total/the only reason** | n = 119/n = 68 (33,8 %) | n = 64/n = 32 (8,6 %) | **0,0075** |
| **- transient increase in total/the only reason** | n = 40/n = 27 (39,7 %) | n = 17/n = 9 (28,1 %) | 0,5040 |
| **- unsatisfactory graft function in total/the only reason** | n = 19/n = 18 (26,5 %) | n = 15/n = 12 (37,5 %) | 0,5300 |
| **group 2 (elevated UACR) in total/the only reason** | n = 59/n = 23 (11,4 %) | n = 38/n = 12 (3,2 %) | 0,4179 |
| **group 3 (DSA positivity) in total/the only reason** | n = 52/n = 18 (9 %) | n = 57/n = 12 (3,2 %) | 0,5040 |
| **group 4 (history of rejection) in total/the only reason** | n = 31/n = 7 (3,5 %) | n = 42/n = 9 (2,4 %) | 0,8995 |
| **group 5 (IS reduction) in total/the only reason** | n = 24/n = 15 (7,5 %) | n = 25/n = 20 (5,4 %) | 0,8030 |
| **group 6 (combined reason)** | n = 70 (34,8 %) | n = 61 (16,5 %) | **0,0180** |
| **follow up measurements in total** | n = 0 | n = 224 (60,5 %) | - |
| **biopsy following dd-cfDNA** | n = 34 (50 %) | n = 48 (13 %) | 0,9034 |
| **- if yes, (months), median, [IQR 25th–75th percentile]** | median 1  IQR 1-5 | median 1  IQR 1-2 | 0,5436 |

SD – Standard deviation, IQR - Interquartile range, DSA – Donor-specific antibodies, dd-cfDNA Donor-derived cell-free DNA – UACR - Urinary albumin-to-creatinine ratio, IS - Immunosuppression

**Table S3** Group characteristics – KTx ≤ 12 months, 12–60 months, > 60 months

| **patients: n = 313**  **measurements: n = 571** | **KTx ≤ 12 months**  **patients: n = 64**  **measurements: n = 115** | **KTx 12-60 months**  **patients: n = 68**  **measurements: n = 136** | **KTx > 60 months**  **patients: n = 181**  **measurements: n = 320** | **P value** |
| --- | --- | --- | --- | --- |
| **sex – male (%)** | n = 46 (71.9 %) | n = 51 (75 %) | n = 112 (61.9 %) | 0,1755 |
| **age at time of kidney transplantation (years) ± SD** | 50.2 ± 14  median 48  min 20, max 84 | 44.1 ± 16  median 44  min 13, max 80 | 40.1 ± 14.6  median 41  min 5, max 71 | **<0,0001** |
| **time after kidney transplantation when dd-cfDNA performed (months), median, [IQR 25th–75th percentile]** | median 5  IQR 2-9 | median 34  IQR 22.8-46 | median 134  IQR 94-191 | **<0,0001** |
| **total cfDNA (cp/mL), median, [IQR 25th–75th percentile]** | median 8244  IQR 4605-17138 | median 4659  IQR 2963-10228 | median 3644  IQR 2412-5944 | **<0,0001** |
| **dd-cfDNA (%), median, [IQR 25th–75th percentile]** | median 0.4  IQR 0.19-1.04 | median 0.34  IQR 0.2-0.73 | median 0.33  IQR 0.2-0.8 | **0,0010** |
| **dd-cf-DNA (cp/mL), median, [IQR 25th–75th percentile]** | median 32  IQR 11-105 | median 15  IQR 9-57 | median 13  IQR 7-34 | **<0,0001** |
| **creatinine base line (mg/dL), median, [IQR 25th–75th percentile]** | median 2  IQR 1.5-2.6 | median 1.74  IQR 1.5-2.3 | median 1.9  IQR 1.6-2.4 | 0,0880 |
| **UACR base line (mg/g), median, [IQR 25th–75th percentile]** | median 49.4  IQR 28.3-132.8 | median 42.3  IQR 15.4-163 | median 151.5  IQR 35.9-523.5 | **<0,0001** |
| **reasons for dd-cfDNA measurement** | | | |  |
| **group 1 (elevated creatinine) in total/the only reason** | n = 46/n = 34 (29.6 %) | n = 39/n = 21(15.4 %) | n = 98/n = 45 (14.1 %) | 0,1167 |
| **- transient increase in total/the only reason** | n = 7/n = 5 (14.7 %) | n = 11/n = 6 (28.6 %) | n = 39/n = 25 (55.6 %) | 0,1512 |
| **- unsatisfactory graft function in total/the only reason** | n = 23/n = 21 (61.8 %) | n = 9/n = 7 (33.3 %) | n = 2/n = 2 (4.4 %) | **<0,0001** |
| **group 2 (elevated UACR) in total/the only reason** | n = 16/n = 7 (6 %) | n = 11/n = 5 (3.7 %) | n = 70/n = 23 (7.2 %) | **0,0010** |
| **group 3 (DSA positivity) in total/the only reason** | n = 9/n = 4 (3.5 %) | n = 26/n = 8 (5.9 %) | n = 74/n = 18 (5.6 %) | **0,0016** |
| **group 4 (history of rejection) in total/the only reason** | n = 12/n = 2 (1.7 %) | n = 16/n = 7 (5.1 %) | n = 45/n = 7 (2.2 %) | 0,6557 |
| **group 5 (IS reduction) in total/the only reason** | n = 3/n = 2 (1.7 %) | n = 14/n = 12 (8.8 %) | n = 32/n = 21 (6.6 %) | **0,0405** |
| **group 6 (combined reason)** | n = 19 (16.5 %) | n = 24 (17.6 %) | n = 88 (27.5 %) | **<0,0001** |
| **follow up measurements in total** | n = 47(40.9 %) | n = 59 (43.4 %) | n = 118 (36.9 %) | 0,3956 |
| **single measurements (%)/multiple measurements (%)** | n = 37 (32.2 %)/n = 78 (67.8 %) | n = 48 (35.3 %)/n = 88 (64.7 %) | n = 116/n = 204 | 0,4272 |
| **biopsy following dd-cfDNA** | n = 20 (17.4 %) | n = 14 (10.3 %) | n = 48 (15 %) | 0,2479 |
| **- if yes, time after measurement (months), median, [IQR 25th–75th percentile]** | median 1  IQR 0.75-4 | median 1  IQR 0-4 | median 1  IQR 0-2.5 | 0,3367 |

SD – Standard deviation, IQR - Interquartile range, DSA – Donor-specific antibodies, dd-cfDNA Donor-derived cell-free DNA – UACR - Urinary albumin-to-creatinine ratio, IS - Immunosuppression

**Table S4** Group characteristics – measurements followed by biopsy

| **patients: n = 58**  **measurements: n = 82** | |
| --- | --- |
| **sex – male (%)** | n = 46 (88.5 %) |
| **age at time of kidney transplantation (years) ± SD** | 44.5 ± 16.4  median 45  min 5, max 80 |
| **time after kidney transplantation when dd-cfDNA performed (months) ± SD** | median 81.5  IQR 14.25-160 |
| **total cfDNA (cp/mL), median, [IQR 25th–75th percentile]** | median 4021  IQR 2721-6402 |
| **dd-cfDNA (%), median, [IQR 25th–75th percentile]** | median 0.44  IQR 0.26-1.5 |
| **dd-cf-DNA (cp/mL), median, [IQR 25th–75th percentile]** | median 21.5  IQR 10.25-55.5 |
| **creatinine base line (mg/dL), median, [IQR 25th–75th percentile]** | median 1.85  IQR 1.53-2.37 |
| **UACR base line (mg/g), median, [IQR 25th–75th percentile]** | median 62.5  IQR 27.5-252 |
| **reasons for dd-cfDNA measurement** | |
| **group 1 (elevated creatinine) in total/the only reason** | n = 39/n = 16 (19.5 %) |
| **- transient increase in total/the only reason** | n = 7/n = 3 (18.8 %) |
| **- unsatisfactory graft function in total/the only reason** | n = 8/n = 8 (50 %) |
| **group 2 (elevated UACR) in total/the only reason** | n = 28/n = 7 (8.5 %) |
| **group 3 (DSA positivity) in total/the only reason** | n = 20/n = 4 (4.9 %) |
| **group 4 (history of rejection) in total/the only reason** | n = 12/n = 3 (3.7 %) |
| **group 5 (IS reduction) in total/the only reason** | n = 2/n = 2 (2.4 %) |
| **group 6 (combined reason)** | n = 35 (42.7 %) |
| **follow-up measurements** | n = 15 (18.3 %) |
| **single/multiple measurements** | n = 58 (70.7 %)/n = 24 (29.3 %) |
| **results of the biopsy** | |
| **TCMR** | n = 6 (7.3 %) |
| **ABMR** | n = 25 (30.5 %) |
| **Mixed rejection** | n = 3 (3.7 %) |
| **borderline** | n = 7 (8.5 %) |
| **recurrence of glomerular disease** | n = 3 (3.7 %) |
| **other** | n = 6 (7,3 %) |
| **negative histology** | n = 32 (39 %) |

**Note:** **TCMR** – class IA (n = 3), class IB (n = 1), class IIA (n = 1), class IIB (n = 1), **recurrence** – IgAN (n = 2), FSGS (n = 1), **other** – IFTA 2-3 (n = 2), BKVN (n = 2), non-representative (n = 2)

SD – Standard deviation, IQR - Interquartile range, DSA – Donor-specific antibodies, dd-cfDNA Donor-derived cell-free DNA – UACR - Urinary albumin-to-creatinine ratio, IS - Immunosuppression

**Table S5** Multivariable linear regression analysis of factors associated with dd-cfDNA (cp/mL)

| **Outcome:** **log-transformed dd-cfDNA (cp/mL)** | **Coefficient (β)** | **95% CI** | **P value** |
| --- | --- | --- | --- |
| **group 1 (elevated creatinine)** | 11,2557 | -1,27164 to 23,7830 | 0,0793 |
| **group 2 (elevated UACR)** | 17,0329 | 5,5427 to 28,5230 | **0,0039** |
| **group 3 (DSA positivity)** | 13,0998 | 2,1800 to 24,0195 | **0,0194** |
| **group 4 (history of rejection)** | 53,4562 | 20,9942 to 85,9182 | **0,0014** |
| **group 5 (IS reduction)** | 6,0567 | -7,81069 to 19,9240 | 0,3927 |
| **group 6 (combined reason)** | -4,0392 | -43,552 to 35,47362 | 0,8413 |
| **follow up measurements** | 33,0751 | 16,2563 to 49,8938 | **0,0001** |

DSA – Donor-specific antibodies, dd-cfDNA Donor-derived cell-free DNA – UACR - Urinary albumin-to-creatinine ratio, IS – Immunosuppression

Models were adjusted for age at transplantation, sex, time post-transplantation at dd-cfDNA measurement, baseline serum creatinine, and baseline UACR. dd-cfDNA (cp/mL) was log-transformed before analysis. Clinical testing indications were entered as binary dummy variables (yes/no), with the absence of the respective indication serving as the reference category. Model statistics: R² = 0.252, adjusted R² = 0.181. No evidence of problematic multicollinearity was observed (all VIFs < 2).

**Table S6** Multivariable linear regression analysis of factors associated with dd-cfDNA (%)

| **Outcome: log-transformeddd-cfDNA (%)** | **Coefficient (β)** | **95% CI** | **P value** |
| --- | --- | --- | --- |
| **group 1 (elevated creatinine)** | 0,386 | 0,265244 to 0,5067 | **<0.0001** |
| **group 2 (elevated UACR)** | 0,5623 | 0,434155 to 0,6904 | **<0.0001** |
| **group 3 (DSA positivity)** | 0,6533 | 0,482682 to 0,8239 | **<0.0001** |
| **group 4 (history of rejection)** | 0,4455 | -0,31165 to 1,2026 | 0,2498 |
| **group 5 (IS reduction)** | 0,3549 | -0,15529 to 0,8650 | 0,1738 |
| **group 6 (combined reason)** | -0,3013 | -0,45549 to -0,1471 | **0,0002** |
| **follow up measurements** | -0,1224 | -0,32467 to 0,0798 | 0,2367 |

DSA – Donor-specific antibodies, dd-cfDNA Donor-derived cell-free DNA – UACR - Urinary albumin-to-creatinine ratio, IS - Immunosuppression

Models were adjusted for age at transplantation, sex, time post-transplantation at dd-cfDNA measurement, baseline serum creatinine, and baseline UACR. dd-cfDNA (%) was log-transformed before analysis. Clinical testing indications were entered as binary dummy variables (yes/no), with the absence of the respective indication serving as the reference category. Model statistics: R² = 0.251, adjusted R² = 0.178. No evidence of problematic multicollinearity was observed (all VIFs < 2).

**3. Supplementary Figures**

|  |  |
| --- | --- |
| elevated creatinine | 183 |
| elevated UACR | 97 |
| DSA positivity | 109 |
| history of rejection | 73 |
| IS reduction | 49 |
| follow up measurement | 224 |

**Figure S1** Reasons for dd-cfDNA measurements – total (whole group)

|  | ABMR | ABMR+TCMR | borderline | TCMR |
| --- | --- | --- | --- | --- |
| in total | 34% | 11% | 15% | 40% |
| the only reason | 19% | 19% | 25% | 37% |

**Figure S2** Types of rejection for “history of rejection” reason of dd-cfDNA measurement

|  | elevated creatinine | elevated UACR | DSA positivity | history of rejection | IS reduction | combined reason | follow up |
| --- | --- | --- | --- | --- | --- | --- | --- |
| single measurement | 33.8 | 11.4 | 9 | 3.5 | 7.5 | 34.8 |  |
| multiple measuremets | 8.6 | 3.2 | 3.2 | 2.4 | 5.4 | 16.5 | 60.5 |

**Figure S3** Reasons for dd-cfDNA measurements – only reasons (single vs. multiple measurements)

**Figure S4** Total cfDNA (cp/ml) – comparison of subgroups based on the reason for the measurement

**Figure S5–S7** Total dd-cfDNA, dd-cfDNA (%), dd-cfDNA (cp/mL) – comparison of multiple vs. single measurement group (log transformation)

**Figure S8–S10** Total dd-cfDNA, dd-cfDNA (%), dd-cfDNA (cp/mL) – comparison of post-transplantation time groups (KTx ≤ 12 m, 12–60 m, > 60 m)

|  | elevated creatinine | elevated UACR | DSA positivity | history of rejection | IS reduction | combined reason | follow up measurements |
| --- | --- | --- | --- | --- | --- | --- | --- |
| KTx ≤ 12 months | 29.6% | 6% | 3.5% | 1.7% | 1.7% | 16.5% | 40.9% |
| KTx 12-60 months | 15.4% | 3.7% | 5.9% | 5.1% | 8.8% | 17.6% | 43.4% |
| KTx > 60 months | 14.1% | 7.2% | 5.6% | 2.2% | 6.6% | 27.5% | 36.9% |

**Figure S11** Reasons for dd-cfDNA measurements – KTx ≤ 12 months, KTx 12-60 months, KTx > 60 months

dd-cfDNA (cp/mL): sensitivity 74.4%, specificity 36.3%, criterion >10, AUC 0.560, p = 0.079

dd-cfDNA (%): sensitivity 65.9%, specificity 49.1%, criterion >0.31%, AUC 0.601, p = 0.003

**Figure S12** Comparative ROC analysis of dd-cfDNA (cp/mL and %) for the prediction of biopsy

|  | elevated creatinine | elevated UACR | DSA positivity | history of rejection | IS reduction | combined reason | follow up |
| --- | --- | --- | --- | --- | --- | --- | --- |
| non-rejection | 25.6% | 15.4% | 2.6% | 7.7% | 2.6% | 33.3% | 12.8% |
| rejection | 14.7% | 2.9% | 8.8% | 0% | 0% | 52.9% | 20.6% |

**Figure S13**  dd-cfDNA measurements followed by biopsy and comparisons between non-rejection histology vs. rejection subgroups

|  |  |
| --- | --- |
| TCMR | 7.3% |
| ABMR | 30.5% |
| mixed rejection | 3.7% |
| borderline | 8.5% |
| recurrence of glomerular disease | 3.7% |
| other | 7.3% |
| no rejection | 39% |

**Note:** **TCMR** – class IA (n = 3), class IB (n = 1), class IIA (n = 1), class IIB (n = 1), **recurrence** – IgAN (n = 2), FSGS (n = 1), **other** – IFTA 2-3 (n = 2), BKVN (n = 2), ATN (n = 1)

**Figure S14** Biopsy results

**Figure S15-17** Total dd-cfDNA, dd-cfDNA (%), dd-cfDNA (cp/mL) – groups comparison of non-rejection histology and rejection subgroups
